# Editorial: Interaction between microbiota and immune in intestinal inflammatory diseases

**DOI:** 10.3389/fcimb.2023.1273282

**Published:** 2023-09-01

**Authors:** Kai Nie, Shiming Yang, Xiaoyan Wang

**Affiliations:** ^1^ Department of Gastroenterology, The Third Xiangya Hospital of Central South University, Changsha, China; ^2^ Hunan Key Laboratory of Nonresolving Inflammation and Cancer, Changsha, China; ^3^ Department of Gastroenterology, Xinqiao Hospital, Third Military Medical University, Chongqing, China

**Keywords:** inflammatory bowel diseases, microbiota, immune, colitis, Kawasaki

Intestinal inflammatory diseases, such as inflammatory bowel disease, systemic vasculitis, infection, and celiac disease, are characterized by complex pathophysiology, wide heterogeneity, and interrelatedness. Inflammatory bowel disease (IBD), which includes ulcerative colitis and Crohn’s disease (CD), is a chronic inflammatory disease of the gastrointestinal tract that is highly disabling, recurrent, and incurable (Chang, 2020). The pathogenesis of IBD is multifactorial and involves genetic and environmental factors including gut microbiota dysbiosis, immune imbalance, infection, and metabolic imbalance (Chang, 2020). Of these factors, microbiota dysbiosis and immune imbalance appear to play significant roles in the development of IBD (Lavelle and Sokol, 2020). Liu et al. perform a quantitative literature analysis of publications in the field of interaction between microbiota and the immune system in intestinal inflammatory diseases, including a total of 3,608 related publications. The authors find that the number of articles in this field has increased annually, and their analysis identifies the authors who have published the most, the most active institutions, and the most active nations. Keyword analysis showed that items such as “regulatory T cell,” “dendritic cell,” and “barrier function” are the most popular in this area. Furthermore, the gut-liver axis and *Fusobacterium nucleatum* have emerged as future research hotspots and trends. This article draws a brief graph of this area, which is consistent with our Research Topic.

For a long time, the study of gut microbiota has been the focus of most studies on intestinal inflammatory diseases, whereas other parts of the flora have been neglected. More importantly, IBD can cause skin inflammation and biological agents used in its mainstream treatment are often associated with serious drug-related skin complications (Rogler et al., 2021). Reiss et al. conducted a study in the Czech Republic that includes 87 patients with IBD and 41 healthy controls, and observe that the skin microbiota signature of patients with IBD differed markedly from that of healthy subjects. In particular, skin microbiota of patients with CD are significantly different to that of patients with ulcerative colitis and healthy subjects. Furthermore, anti-TNF-related skin adverse events are associated with specific shifts in skin microbiota profiles, as well as with decreased serum levels of L-FABP and I-FABP, in patients with IBD. Changes in skin flora may involve the gut–skin axis, including the migration of immune cells and the exchange of cytokines between the intestinal and skin tissues. This study addresses the neglected role of skin microbiota in IBD and provides new insights into IBD-related skin complications.

In addition, IBD can be complicated by severe infection with *Clostridium difficile* resulting from antibiotic use, which is common in patients with IBD (Lamb et al., 2019). Fecal microbiota transplantation (FMT) has proven to be effective in treating *Clostridium difficile* infection (CDI) (Lamb et al., 2019); however, few studies have focused on microbiota shifts in IBD patients with CDI. A study conducted by Yu et al. in China reports that patients with IBD combined with CDI had an abnormal microbiota, mainly varying in terms of the fungi, but the difference between patients with IBD and healthy subjects is much smaller. Moreover, abnormal microbiota and gut glycopeptide synthesis may further affect immunity and promote CDI events. These findings provide new evidence for the treatment of IBD with CDI.

Specific gut bacteria play essential roles in immune modulation and IBD. *Yersinia enterocolitica* is a zoonotic bacterium that exists widely in nature and is one of the most common causes of acute infectious enterocolitis. The clinical manifestations of this disease are similar to those of CDI (Le Baut et al., 2018). Fang et al. discuss the current knowledge of the association of enterocolitic bacteria and their derived microbial compounds with CD pathogenesis. *Y. enterocolitica* infection induces the long-term reprogramming of immune cells and evokes immune dysfunction and gut microbiota dysbiosis, leading to the development of CD in genetically susceptible individuals. Innovative biological drugs based on *Y. enterocolitica*, genetically engineered bacteria, and vaccines are potential therapeutic options for the treatment of CD.

Oncological studies have provided extensive evidence that gut microbiota affect the success of immunotherapy. Moreover, the mechanism underlying the role of microbiota in immunotherapy directly influences immune state. Lv et al. find that *Lactobacillus plantarum* enhances the inhibitory effect of tacrolimus on colitis; specific mechanisms include the interferon pathway, graft resistance, and the IL-2 signaling pathway, which affect intestinal bacteria and improve bile acid metabolism. This provides direct evidence for the influence of gut microbiota on intestinal immune function regulated by immunotherapy (Wallace et al., 2014). In addition, it provides a basis for the individualized application of immunosuppression-related drugs in the mechanism of intestinal inflammatory diseases.

Kawasaki disease, a systemic vasculitis, can also affect the intestines, causing inflammatory damage such as ulcers and loss of mucosal integrity. Wang et al. observe a decrease in gut butyric acid-producing bacteria in a mouse model of Kawasaki disease. They further find that butyric acid improves intestinal barrier function, significantly reduces inflammatory reactions, and ameliorates vascular damage. Butyrate can dephosphorylate activated JNK, ERK1/2, and p38 MAPK in RAW264.7 macrophages by increasing the expression of phosphatase MKP-1, which exhibits significant anti-inflammatory effects. This study provides evidence that bacterial metabolites influence immune cells and maintain gut barrier function.

In conclusion, the articles in this Research Topic present the latest developments in intestinal inflammatory diseases, providing new insights into the interactions between the gut microbiota and immunity in IBD, Kawasaki disease, and colitis. Each study presents new threads and questions worth exploring and elucidating. Although the understanding of the mechanism of interaction between microbiota and immunity in intestinal inflammatory diseases has advanced significantly in recent years, there are still many ignored concepts and a lack of effective microbiota-immune therapy. Hence, this Research Topic aims to shed light on these concepts and advance our understanding of the subject.

## Author contributions

KN: Writing – original draft. SY: Conceptualization, Writing – review & editing. XW: Conceptualization, Supervision, Writing – review & editing.

## References

[B1] Chang J. T. (2020). Pathophysiology of inflammatory bowel diseases. N Engl. J. Med. 383, 2652–2664. doi: 10.1056/NEJMra2002697 33382932

[B2] Lamb C. A. Kennedy N. A. Raine T. Hendy P. A. Smith P. J. Limdi J. K.. (2019). British Society of Gastroenterology consensus guidelines on the management of inflammatory bowel disease in adults. Gut 68, s1–s106. doi: 10.1136/gutjnl-2019-318484 31562236 PMC6872448

[B3] Lavelle A. Sokol H. (2020). Gut microbiota-derived metabolites as key actors in inflammatory bowel disease. Nat. Rev. Gastroenterol. Hepatol. 17, 223–237. doi: 10.1038/s41575-019-0258-z 32076145

[B4] Le Baut G. O'Brien C. Pavli P. Roy M. Seksik P. Treton X.. (2018). Prevalence of yersinia species in the ileum of Crohn's disease patients and controls. Front. Cell Infect. Microbiol. 8, 336. doi: 10.3389/fcimb.2018.00336 30298122 PMC6160741

[B5] Rogler G. Singh A. Kavanaugh A. Rubin D. T. (2021). Extraintestinal manifestations of inflammatory bowel disease: current concepts, treatment, and implications for disease management. Gastroenterology 161, 1118–1132. doi: 10.1053/j.gastro.2021.07.042 34358489 PMC8564770

[B6] Wallace K. L. Zheng L. B. Kanazawa Y. Shih D. Q. (2014). Immunopathology of inflammatory bowel disease. World J. Gastroenterol. 20, 6–21. doi: 10.3748/wjg.v20.i1.6 24415853 PMC3886033

